# De Bruijn Superwalk with Multiplicities Problem is NP-hard

**DOI:** 10.1186/1471-2105-14-S5-S7

**Published:** 2013-04-10

**Authors:** Evgeny Kapun, Fedor Tsarev

**Affiliations:** 1 St. Petersburg National Research University of Information Technologies, Mechanics and Optics Genome Assembly Algorithms Laboratory 197101, Kronverksky pr., 49, St. Petersburg, Russia

## Abstract

De Bruijn Superwalk with Multiplicities Problem is the problem of finding a walk in the de Bruijn graph containing several walks as subwalks and passing through each edge the exactly predefined number of times (equal to the multiplicity of this edge). This problem has been stated in the talk by Paul Medvedev and Michael Brudno on the first RECOMB Satellite Conference on Open Problems in Algorithmic Biology in August 2012. In this paper we show that this problem is NP-hard. Combined with results of previous works it means that all known models for genome assembly are NP-hard.

## Introduction

The majority of current genome sequencing technologies are based on the shotgun method -- the genome is split into several small fragments which are read directly. The problem of reconstructing the initial genome from these small fragments (reads) is known as the genome assembly problem. It is one of the fundamental problems of bioinformatics. Several models for genome assembly were studied by researchers. If reads are assumed to be error-free, the assumption made in all models is that every read from the input must be a substring of the genome.

One of the models is based on maximum parsimony principle -- the original genome should be the shortest string containing all reads as substrings. This leads to the Shortest Common Superstring (SCS) problem which is NP-hard [1]. Modeling genome assembly as the SCS problem has a sufficient drawback: the majority of genomes have repeats -- multiple similar (or even equal) fragments, while the SCS solution would under-represent these repeats.

The de Bruijn graph model proposed in [2] deals with repeats much better. It is based on generating a set of all (*k *+ 1)-character substrings (called (*k *+ 1)-mers) of reads and constructing a de Bruijn graph in which the vertices are *k*-mers and edges are (*k *+ 1)-mers. Each read is represented by a walk in this graph. Any walk containing all the reads as subwalks represents a valid assembly. Consequently, the genome assembly problem is formulated as finding the shortest superwalk, which is closely related to the polynomial time Eulerian tour problem (which was previously used to solve the problem of sequencing by hybridization [3]). Despite that, the Shortest De Bruijn Superwalk problem (SDBS) was shown to be NP-hard [4]. Note also that SDBS has a special case solvable in polynomial time -- if each subwalk contains only one edge, this problem can be reduced to Chinese Postman Problem [5].

In [6] an algorithm for reads' copy counts estimation based on maximum likelihood principle was proposed. A similar algorithm can be applied to find multiplicities of edges in the de Bruijn graph, so, the following problem was formulated in the talk [7]. Given a de Bruijn graph with vertices of size *k *constructed from a set of reads and multiplicities (in unary notation) of all edges of this graph find a superwalk consistent with edge multiplicities and containing all reads as subwalks. This problem is named De Bruijn Superwalk with Multiplicities problem (DBSM) and its computational complexity remained unknown.

In this paper we prove that this problem is NP-hard.

## NP-hardness proof

The proof has the following structure. First, the Common Superstring with Multiplicities (CSM) problem is formulated. This problem is shown to be NP-hard by reducing SCS to it. Then CSM is reduced to de Bruijn Superwalk with Multiplicities problem.

Let *S *be a string over alphabet *∑*. Let *L_c_* (*S*) denote the number of occurrences of character *c *∈ *∑ *in *S*. Then, let Common Superstring with Multiplicities problem be the problem, given strings *S*_1_, *S*_2_, ..., *S_n_* and nonnegative integers *l_c_* for all *c *∈ *∑ *(given in unary notation), to find out if there exists a string *S *such that:

- all strings *S*_1_, *S*_2_, ..., *S_n_* are substrings of *S*,

- *L_c_* (*S*) = *l_c_* for each *c *∈ *∑*.

**Theorem 1**. *Common Superstring with Multiplicities problem is NP-hard for *|*∑*| = 2.

*Proof*. To prove this, we take an instance of Shortest Common Superstring problem with *∑ *= {0, 1}, which is NP-hard [8], and transform it into an instance of Common Superstring with Multiplicities problem with the same answer. Let the original instance of SCS problem be $\left\{S_{1}^{\prime },S_{2}^{\prime },...,S_{n}^{\prime }\right\}$, *l*' (this instance means that we need to find if there exists a superstring of $S_{1}^{\prime },S_{2}^{\prime },...,S_{n}^{\prime }$ having length at most *l*').

Let us define *T*_0_ = 000111 and *T*_1_ = 001011. These strings have been selected in such a way that each of them contains the same number of zeroes and ones and they do not overlap -- no proper suffix of any of the *T_c_*(*c *∈ {0, 1}) is equal to any of the proper prefixes of any of the *T_c_*(*c *∈ {0, 1}).

Then, let $S_{k}=T\left(S_{k}^{\prime }\right)$ and *l*_0_ = *l*_1_ = 3*l*', where $T\left(c_{1}c_{2}\;\;\ldots \;\;c_{k}\right)=T_{c_{1}}T_{c_{2}}\;\;\ldots \;\;T_{c_{k}}$. The following lemmas formulate several properties of these instances of SCS and CSM problems. Equivalence of these instances is shown in lemmas 3 and 7.

**Lemma 1**. *L*_0_(*T*(*S*')) = *L*_1_(*T*(*S*')) = 3|*S*'|.

*Proof*. It follows directly from the definition of *T*.

**Lemma 2**. *If $S_{1}^{\prime }$is a substring of $S_{2}^{\prime }$, then $T\left(S_{1}^{\prime }\right)$ is a substring of $T\left(S_{2}^{\prime }\right)$*.

*Proof*. It follows directly from the definition of *T*.

**Lemma 3**. *If the answer for the original instance of SCS problem is positive, then the answer for the instance of CSM problem is also positive*.

*Proof*. If the answer for the instance of SCS problem is positive, then there exists a string *S*' of length *l*'' ≤ *l*' such that *S*' is a superstring of $S_{1}^{\prime },S_{2}^{\prime },\ldots ,S_{n}^{\prime }$. Then, let *S *= *T*(*S*'0^*l*'-*l*''^). Because |*S*'0^*l*'-*l*''^| = |*S*'| + |0^*l*'-*l*''^| = *l*'' + (*l*'-*l*'') = *l*', *L*_0_(*S*) = *L*_1_(*S*) = 3*l*' (see lemma 1) and all *S_i_*are substrings of *T*(*S*') (see lemma 2) the answer to the instance of CSM is indeed positive.

**Lemma 4**. *Let $S_{1}^{\prime }$and $S_{2}^{\prime }$be two strings such that there is a suffix of $T\left(S_{1}^{\prime }\right)$ equal to a prefix of $T\left(S_{2}^{\prime }\right)$. Then the following holds:*

- *the length of that suffix is a multiple of *6,

- *if the length of the suffix is *6*k, then the suffix of length k of $S_{1}^{\prime }$ is equal to the prefix of length k of $S_{2}^{\prime }$*.

*Proof*. Suppose that the length of the suffix is equal to 6*k *+ *i*, 0 *< i <*6. Let *c*_1_ be the last character of $S_{1}^{\prime }$ and *c*_2_ be the character at the (*k *+ 1)-th position of $S_{2}^{\prime }$ (positions are numbered starting from one). Then, the suffix of $T_{c_{1}}$ of length *i *would be equal to the prefix of $T_{c_{2}}$ of the same length.

As mentioned before, no proper suffix of any of the *T_c_*(*c *∈ {0, 1}) is equal to any of the proper prefixes of any of the *T_c_*(*c *∈ {0, 1}). Therefore, the length of the suffix is a multiple of 6. The second follows from *T*_0_ and *T*_1_ both having length 6 and *T*_0_ ≠ *T*_1_.

**Lemma 5**. *Let $S_{1}^{\prime }$and $S_{2}^{\prime }$ be two strings such that $T\left(S_{1}^{\prime }\right)$ is a substring of $T\left(S_{2}^{\prime }\right)$*.

Then following statements hold:

- *each occurrence of $T\left(S_{1}^{\prime }\right)$ in $T\left(S_{2}^{\prime }\right)$ starts at a position which is congruent to *1 *modulo *6,

- *if $T\left(S_{1}^{\prime }\right)$ occurs at position *6*k *+ 1 *in $T\left(S_{2}^{\prime }\right)$, then $S_{1}^{\prime }$ occurs as a substring of $S_{2}^{\prime }$ at position k *+ 1.

*Proof*. The proof is analogous to lemma 4.

**Lemma 6**. *Let $S_{1}^{\prime },S_{2}^{\prime },...,S_{n}^{\prime }$ be a set of strings, and let S be a superstring of $T\left(S_{1}^{\prime }\right)$*, $T\left(S_{2}^{\prime }\right),\ldots ,T\left(S_{n}^{\prime }\right)$*such that $T\left(S_{1}^{\prime }\right)$*, $T\left(S_{2}^{\prime }\right),...,T\left(S_{n}^{\prime }\right)$*occur in S at positions i*_1_, *i*_2_, ..., *i_n_ respectively (if some $T\left(S_{k}^{\prime }\right)$ occurs in S in multiple positions only one position is recorded) and every character of S is covered by at least one of those occurrences. Then the following statements hold:*

- *i*_1_, *i*_2_, ..., *i_n_ are all congruent to *1 *modulo *6,

- *length of S is a multiple of *6,

- *There exists a string S*' *such that S *= *T*(*S*')*. Strings $S_{1}^{\prime },S_{2}^{\prime },...,S_{n}^{\prime }$ occur in S*' *at positions $i_{1}^{\prime },i_{2}^{\prime },...,i_{n}^{\prime }$,where $i_{k}=6i_{k}^{\prime }-5$ for k *= 1, 2, ..., *n*.

*Proof*. Suppose the contrary. Let *i_k_*be the smallest of *i*_1_, *i*_2_, ..., *i_n_*which is not congruent to 1 modulo 6. Then, if *i_k_*-th character of *S *is covered by some $T\left(S_{k^{\prime }}^{\prime }\right)$ such that *i*_*k*'_ <*i_k_*, we have a contradiction because *i*_*k*' _is not congruent with *i_k _*modulo 6, but either $T\left(S_{k}^{\prime }\right)$ and $T\left(S_{k^{\prime }}^{\prime }\right)$ overlap, or $T\left(S_{k}^{\prime }\right)$ is a substring of $T\left(S_{k^{\prime }}^{\prime }\right)$, which would violate either lemma 4 or lemma 5. If *i_k_*-th character of *S *is not covered by any $T\left(S_{k^{\prime }}^{\prime }\right)$, such that, *i*_*k*' _ <*i_k_*, than (*i_k_*- 1)-th character of *S *must be covered by the last character of some $T\left(S_{k^{\prime }}^{\prime }\right)$. But length of $T\left(S_{k^{\prime }}^{\prime }\right)$ is a multiple of 6, so *i_k_*must be congruent to *i*_*k*' _modulo 6, which leads to a contradiction.

The last character of *S *is also covered by the last character of some $T\left(S_{k}^{\prime }\right)$. Because *i_k_*is congruent to 1 modulo 6 and the length of $T\left(S_{k}^{\prime }\right)$ is a multiple of 6, the length of *S *is also a multiple of 6.

To prove the last point, it is enough to notice that for *j *= 1, 7, ..., |*S*| - 5, the substring of *S *starting at position *j *and having length 6 is either *T*_0_ or *T*_1_. This follows from the fact that the *j*-th character of *S *is covered by an occurrence of $T\left(S_{k}^{\prime }\right)$ for some *k*, but restrictions on lengths of $T\left(S_{k}^{\prime }\right)$ and on *i_k_*mean that the whole substring of length 6 would be covered by $T\left(S_{k}^{\prime }\right)$. Moreover, the position at which the substring of length 6 occurs in $T\left(S_{k}^{\prime }\right)$ is congruent to 1 modulo 6, therefore that substring is either *T*_0_ or *T*_1_ by definition of *T *.

**Lemma 7**. *If the answer for the instance of CSM problem is positive, then the answer for the original instance of SCS problem is also positive*.

*Proof*. If the answer for the instance of CSM problem is positive, then there exists a string *S *of length 6*l*' which is a superstring of *S*_1_, *S*_2_, ..., *S_n_*. Let *S*'' be the shortest common superstring of these strings. Then |*S*''| ≤ 6*l*' and each character of *S*'' is covered by an occurrence of one of *S*_1_, *S*_2_, ...,*S_n_*. Recall that $S_{k}=T\left(S_{k}^{\prime }\right)$. By lemma 6, there exists a string *S*' such that *S*'' = *T*(*S*') and $S_{1}^{\prime },S_{2}^{\prime },...,S_{n}^{\prime }$ are substrings of *S*'. Also the equation $|S^{\prime }|=\frac{\left|S^{\prime \prime }\right|}{6}\leq \frac{6l^{\prime }}{6}=l^{\prime }$ holds. Therefore, the answer for the original instance of SCS problem is also positive.

**Theorem 2**. *The de Bruijn Superwalk with Multiplicities Problem is NP-hard for any fixed *|*∑*| ≥ 2 *and any positive integer k*.

**Figure 1:**
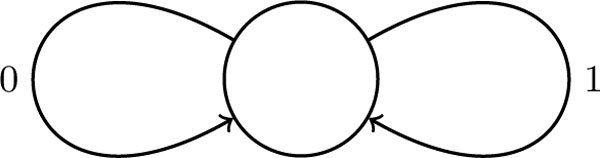
**A graph on which Common Superwalk with Multiplicities problem is NP-hard**.

*Proof*. Consider the graph with one vertex and two loops (see Figure 1). An instance of Common Superstring with Multiplicities problem with *∑ *= {0, 1} can be translated into an instance of Superwalk with Multiplicities problem on this graph in the following way:

- each *S_k_* is directly translated into a walk, by representing 0 as occurrence of edge 0 and 1 as occurrence of edge 1 in the walk,

- the multiplicity of edge 0 is set to *l*_0_, and the multiplicity of edge 1 is set to *l*_1_.

To complete the proof we need to embed this graph into a de Bruijn graph with given *k*.

**Figure 2:**
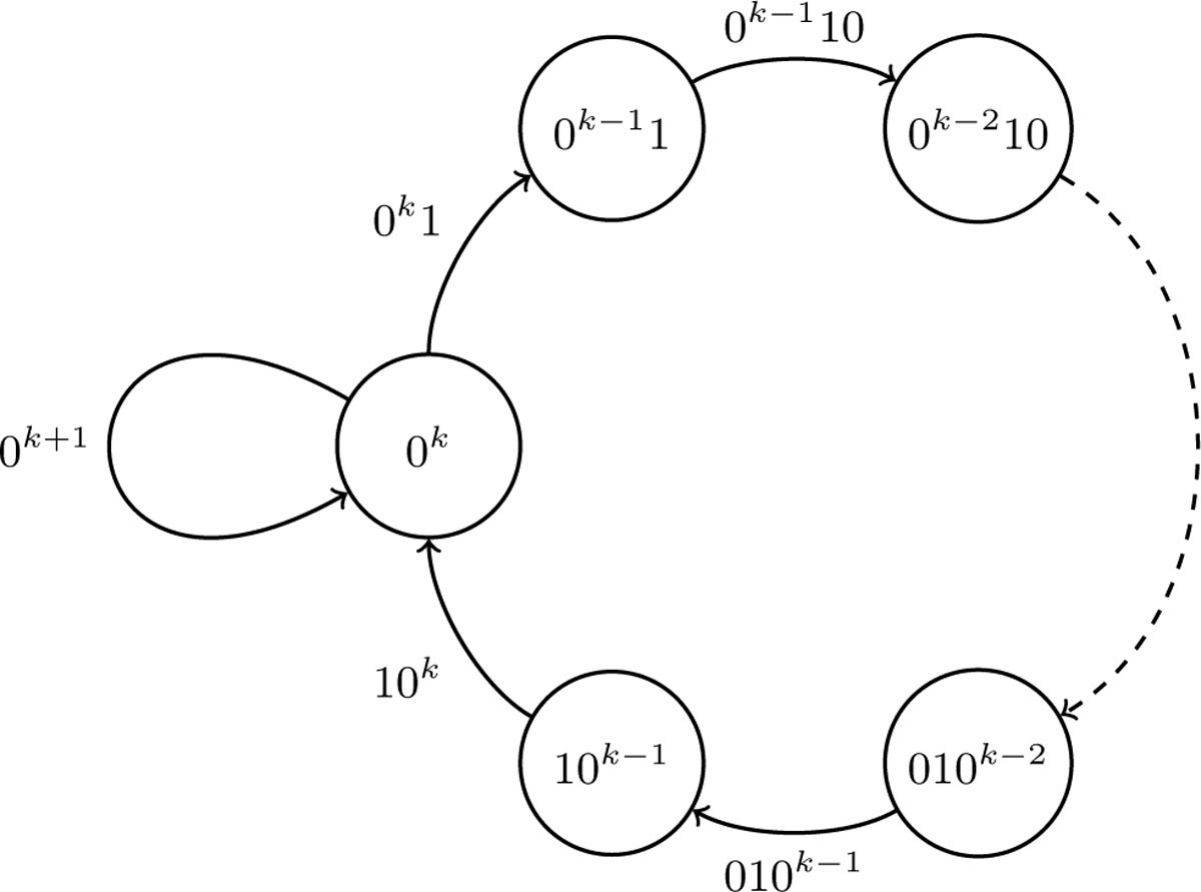
**Embedding of the graph from the figure 1 into a de Bruijn graph**.

This can be done in straightforward manner (see Figure 2). Edge 0 is mapped to a loop, while edge 1 is mapped to a cycle of length *k *+ 1.

## Conclusion

We have proved that the de Bruijn Superwalk with Multiplicities Problem is NP-hard. Results of this work combined with [4] show that all known models for genome assembly are NP-hard.

However, both de Bruijn Shortest Superwalk and de Bruijn Superwalk with Multiplicities problems have a special case (if subwalks consist of one edge) solvable in polynomial time. A reasonable direction for future research is to find if there exist other polynomially solvable special cases of these problems.

## Authors' contributions

The work presented here was carried out in collaboration between all authors. All authors have contributed to, seen and approved the manuscript.

## Declarations

Publication of this article was supported by the Ministry of Education and Science of Russian Federation in the framework of the federal program "Scientific and scientific-pedagogical personnel of innovative Russia in 2009-2013" and by the University ITMO.

This article has been published as part of *BMC Bioinformatics *Volume 14 Supplement 5, 2013: Proceedings of the Third Annual RECOMB Satellite Workshop on Massively Parallel Sequencing (RECOMB-seq 2013). The full contents of the supplement are available online at http://www.biomedcentral.com/bmcbioinformatics/supplements/14/S5.
